# Loss of Sirtuin 1 (SIRT1) potentiates endothelial dysfunction via impaired glycolysis during infectious challenge

**DOI:** 10.1002/ctm2.1054

**Published:** 2022-09-14

**Authors:** Ryan J. Stark, Stephen R. Koch, Cody L. Stothers, Allison Pourquoi, Celia K. Lamb, Michael R. Miller, Hyehun Choi

**Affiliations:** ^1^ Department of Pediatrics Vanderbilt University Medical Center Nashville Tennessee

## Dear editor

Sepsis is an exaggerated host response to infection that despite advances in early recognition and intervention, remains a common cause of morbidity and mortality.^1^, ^2^ The pathophysiology is related to global inflammation and cytokinemia driving endothelial dysfunction to produce the prototypic signs of vasculopathy such as altered vasomotor tone and capillary leak.^3^, ^4^, ^5^ Silent information regulator 2 (Sir2) proteins (sirtuins), are a class of seven NAD‐dependent deacetylases or ADP‐ribosyltransferases that regulate cellular homeostasis.^6^ Sirtuin 1 (SIRT1) is the best characterised, specifically related to cellular metabolism.^7^ The effects of SIRT1 have been postulated to be mediated by endothelial nitric oxide synthase (eNOS), forkhead box O1(FOXO1), peroxisome proliferator‐activated receptor‐γ coactivator 1α (PGC1α) and mitochondrial transcription factor A (TFAM). Thus, perturbations in SIRT1 during sepsis may impair metabolism and directly impact endothelial function.

**FIGURE 1:**
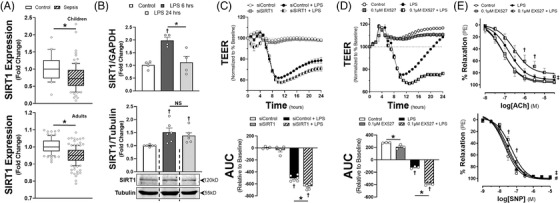
(A) SIRT1 mRNA relative values on day 0 (<24 h) of hospitalisation are shown for children (*top*, ≤10 years of age) or adult (*bottom*, >18 years of age) patients with or without sepsis from the respective GEO Datasets (GSE26378, children and GSE134364, adults). The median expression value is shown with a horizontal bar per condition with 75% interquartile range normalised to the respective controls (children: controls *n* = 21, sepsis *n* = 79; adults: controls *n* = 83, sepsis *n* = 156). (B) qRT‐PCR values of SIRT1 mRNA normalised to GAPDH control (*top*, *n* = 4 individual replicates) or SIRT1 protein expression with associated western blots normalised to tubulin (*bottom*, *n* = 6 individual replicates) either with vehicle or LPS treatment (100 ng/ml) for 6 or 24 h in HMVECs. (C) *Top*: Transendothelial electrical resistance (TEER) of HMVECs treated with scrambled siRNA (siControl) or siRNA to SIRT1 for 72 h prior to LPS exposure of 100 ng/ml and subsequently followed for 24 h. Data are normalised to baseline prior to LPS exposure. Open circles represent HMVECs exposed to siControl for 72 h without LPS (vehicle). *Bottom*: Area under the curve (AUC) relative to change from baseline is shown for the TEER. †*p* < .05 between indicated group and control. *p < .05 between designated groups. *n* = 4–6 individual replicates per group. (D) *Top*: TEER of HMVECs pretreated with 0.1 μM EX527 30 min prior to LPS compared to vehicle control. All data are normalised to baseline resistance per condition prior to addition of EX527. *Bottom*: AUC relative to change from baseline resistance over time calculated for all conditions following EX527 or vehicle addition out to 24 h. †*p* < .05 between indicated group and control. *p < .05 between designated groups. *n* = 3 individual replicates per group. (E) Relaxation of isolated murine mesenteric arteries to escalating doses of acetylcholine (ACh, *top*) or sodium nitroprusside (SNP, *bottom*) treated with control (DMSO, *n* = 8), EX527 (0.1 μM, *n* = 8), LPS (100 ng/ml, *n* = 7) or EX527 with LPS (*n* = 8) for 24 h prior to myography assessments. †*p* < .05 between LPS and EX527 + LPS at the respective dose. #*p* < .05 for EC50 between LPS and EX527 + LPS. ‡*p* < .05 for EC50 between EX527 and EX527 + LPS

**FIGURE 2:**
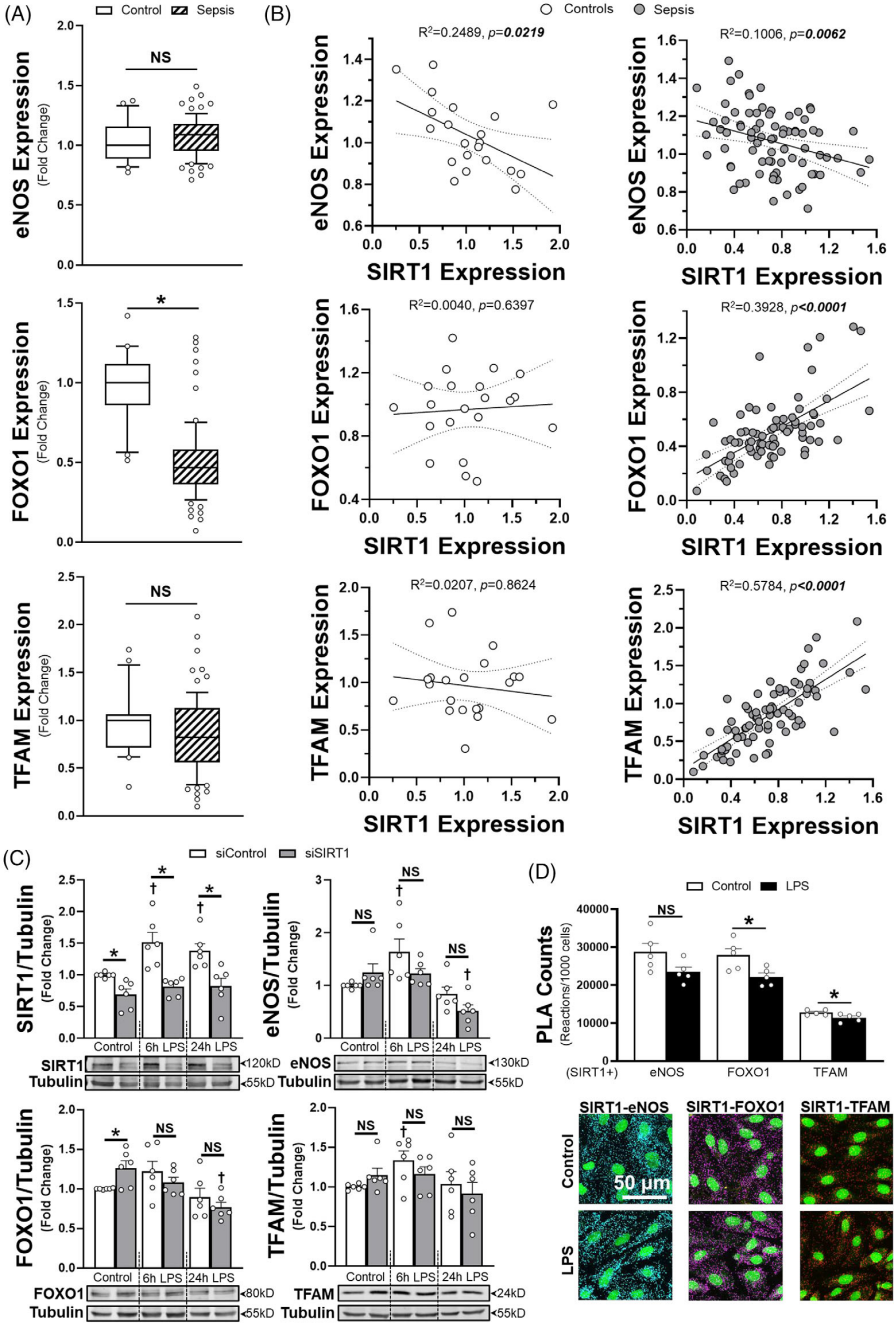
(A) eNOS, FOXO1 and TFAM mRNA transcript relative values are shown for children with or without sepsis from the respective GEO Dataset (GSE26378). The median expression value is shown with a horizontal bar per condition with 75% interquartile range normalised to the respective cohort controls. (B) Expression values for eNOS, FOXO1 or TFAM in comparison to individually matched SIRT1 transcript values for control patients or patients with sepsis. Associated coefficients of determination (*R* ^2^) are shown with Spearman r correlation coefficient *p* values. Linear regression (solid lines) with 95% CI (dotted lines) are displayed. *p < .05 between indicated group and control cohort. NS = non‐significant. (C) Densitometry analysis and associated images of whole cell lysate western blots (*n* = 6 individual replicates per group) calculated for SIRT1, eNOS, FOXO1 or TFAM expression relative to tubulin. HMVECs were treated with respective siRNA for 72 h prior to LPS exposure (100 ng/ml) at the times indicated. (D) Proximity ligation assay (PLA) counts normalised to cell number for SIRT1 interactions with eNOS, FOXO1 and TFAM with or without LPS exposure for 6 h. Associated representative confocal images (40×, line bar represents a distance of 50 μm) are shown for SIRT1‐eNOS (cyan), SIRT1‐FOXO1 (purple) and SIRT1‐TFAM (red) PLA reactions. *n* = 5 individual replicates per group. *p < .05 between designated groups. †*p* < .05 between indicated group and respective siRNA control. NS = non‐significant

To test this hypothesis, we first examined two transcriptomic data sets from the GEO database (GSE26378, children and GSE134364, adults). Sepsis reduced SIRT1 expression in an age‐dependent manner (Figure 1A, Supplementary Figure S1), a finding similar to isolated mononuclear cells exposed to lipopolysaccharide (LPS).^8^ In contrast, in human microvascular endothelial cells (HMVECs) exposed to Gram‐negative LPS, SIRT1 expression increased in a time‐dependent manner (Figures 1B and 2C). To explore if expression was protective, we applied EX527, a SIRT1 inhibitor or siRNA to SIRT1 (siSIRT1) prior to LPS. EX527 or siSIRT1 reduced transendothelial electrical resistance (TEER), a surrogate of endothelial permeability (Figure 1C and D). We also applied EX527 to *ex vivo* LPS‐treated mesenteric arteries. Application impaired relaxation to both acetylcholine (ACh) and sodium nitroprusside (SNP, Figure 1E). Alternatively, SIRT1 knockdown had variable impact on endothelial cytokine production (Supplementary Figure S2).

Next, we correlated SIRT1 transcription in human septic patients (GEO Dataset GSE26378) to expression of eNOS, FOXO1, PGC1α and TFAM, known mediators of SIRT1‐dependent effects. eNOS, FOXO1 and TFAM all showed significant correlation with SIRT1 expression in whole blood, though only FOXO1 was altered in sepsis (Figure 2A and B). As blood cell proteins may not correlate with those in endothelial cells, we determined how siSIRT1 impacted eNOS, FOXO1 and TFAM protein abundance in HMVECs. For all three proteins, there was a time‐dependent reduction in protein after LPS in siSIRT1 HMVECs compared to siRNA controls (siControl), with the largest impacts in eNOS and FOXO1 expression at 24 h (Figure 2C). Proximity ligation assays (PLA) and immunoprecipitation revealed that SIRT1 had direct interactions with all three proteins, though the amount of interaction was significantly higher with eNOS and FOXO1 compared to TFAM (Figure 2D, Supplementary Figure S3). Application of LPS for 6 h induced a significant, albeit small reduction in SIRT1 interaction with FOXO1 and TFAM. Mitogen‐activated protein kinase signalling, which drives endothelial activation and cytokine production, was not significantly different between siControl and siSIRT1 treated cells (Supplementary Figure S4). In addition, despite prior known associations, PGC1α transcript levels in whole blood and protein expression in HMVECs demonstrated no relationship with SIRT1(Supplementary Figure S5).

**FIGURE 3:**
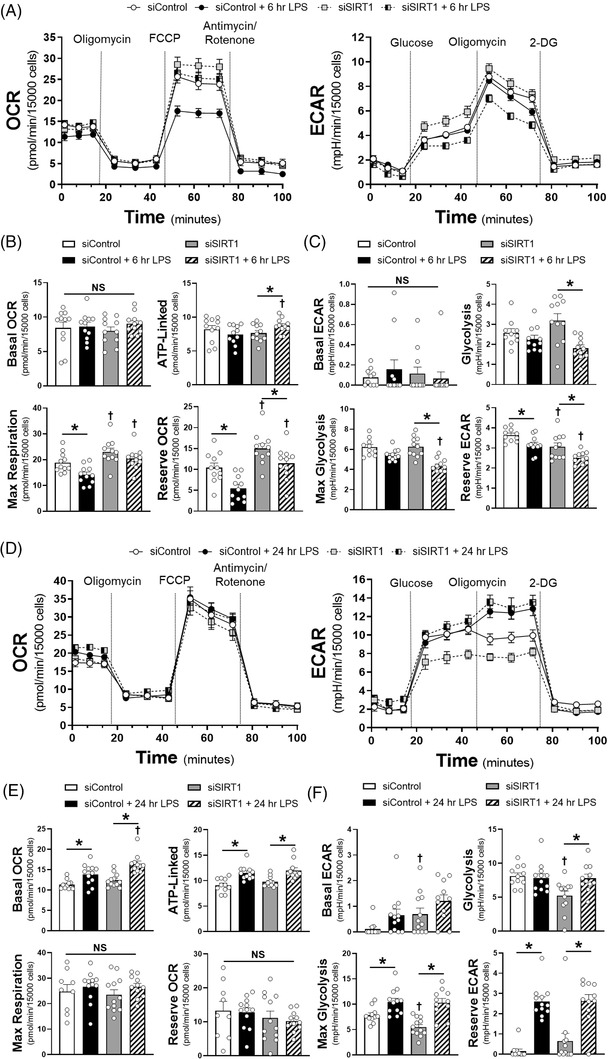
(A) Seahorse XFe96 assays for oxygen consumption rate (OCR, *left*) or extracellular acidification rate (ECAR, *right*) using Control and SIRT1 siRNA treated HMVECs with 6 h of LPS exposure (100 ng/ml) prior to assays. (B) Respective components of OCR via calculation as follows: ‘Basal OCR’ = pre Oligomycin – post Antimycin/Rotenone, ‘ATP‐linked’ = pre Oligomycin – post Oligomycin, ‘Max Respiration’ = post FCCP – post Antimycin/Rotenone, ‘Reserve OCR’ = post FCCP – pre FCCP. (C) Respective ECAR components with calculations as follows: ‘Basal ECAR’ = pre Glucose – post 2‐deoxyglucose (2‐DG), ‘Glycolysis’ = post Glucose – pre Glucose, ‘Max Glycolysis’ = post Oligomycin – post 2‐DG, ‘Reserve ECAR’ = post Oligomycin – pre Oligomycin. *n* = 11‐12 individual replicates per group. (D) Seahorse XFe96 assays for oxygen consumption rate (OCR, *left*) or extracellular acidification rate (ECAR, *right*) using Control and SIRT1 siRNA treated HMVECs with 24 h of LPS exposure or condition media prior to assays. Components of OCR (E) or ECAR (F) for respective siRNA ECs treated with or without 24 h of LPS exposure. *p < .05 between designated groups. †*p* < .05 between indicated group and respective siRNA control

**FIGURE 4:**
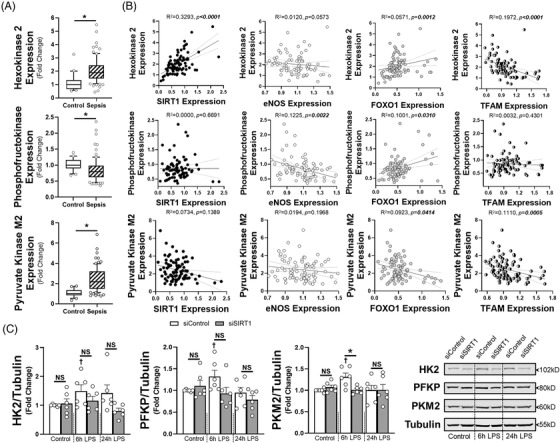
(A) Hexokinase 2 (HK2), phosphofructokinase (platelet, PFKP) or pyruvate kinase M2 (PKM2) mRNA transcript relative values are shown for children with or without sepsis from the respective GEO Dataset (GSE26378). The median expression value is shown with a horizontal bar per condition with 75% interquartile range normalised to the respective cohort controls. (B) Expression values for HK2, PFKP or PKM2 in relation to individually matched transcript levels for SIRT1, eNOS, FOXO1 or TFAM for patients with sepsis. Associated coefficients of determination (*R* ^2^) are shown with Spearman r correlation coefficient *p* values. Linear regression (solid lines) with 95% CI (dotted lines) are displayed. *p < .05 between indicated group and control cohort. NS = non‐significant. (C) Densitometry analysis (*left*) and associated images (*right*) of whole cell lysate western blots (*n* = 6 individual replicates per group) calculated for HK2, PFKP or PKM2 expression relative to tubulin. HMVECs were treated with respective siRNA for 72 h prior to LPS exposure (100 ng/ml) at the times indicated. *p < .05 between designated groups. †*p* < .05 between indicated group and respective siRNA control. NS = non‐significant

To determine the impact of SIRT1 on endothelial metabolism, we utilised the Seahorse metabolic assay to examine both mitochondrial coupling (oxygen consumption rate, OCR) and glycolysis (extracellular acidification rate, ECAR) at 6 and 24 h of LPS exposure. At 6 h there was suppression of maximal respiratory capacity in the siControl HMVECs exposed to LPS, while glycolysis was impaired in siSIRT1 HMVECs exposed to LPS (Figure 3A–C). By 24 h, basal and ATP‐linked oxygen consumption we higher in LPS treated cells, but all cells had the same respiratory capacity after FCCP‐induced uncoupling (Figure 3D and E). In addition, while the impact on glycolysis was similar in control and SIRT1 knockdown ECs exposed to 24 h of LPS, glycolysis was lower in siSIRT1 cells exposed to condition media for 24 h, suggesting that loss of SIRT1 impaired glycolysis dynamically (Figure 3F). To explore the mechanism by which SIRT1 impacted metabolism, we examined whole blood transcript levels for hexokinase 2 (HK2), phosphofructokinase‐platelet (PFKP), and pyruvate kinase M2 (PKM2), rate limiting enzymes in glycolysis, as well as cytochrome C and ATP synthase, rate limiting steps in oxidative phosphorylation. Septic patients had enhanced transcription of HK2 and PKM2 and suppression of PFKP and ATP synthase (Figure 4A, Supplementary Figure S6A). SIRT1 expression strongly correlated with HK2 and cytochrome C, while nearly all metabolic enzymes correlated with FOXO1, and to some extent, TFAM and eNOS (Figure 4B, Supplementary Figure S6B). To test the impact of SIRT1 knockdown on endothelial glycolytic enzymes, HMVECs were exposed to siRNA and then LPS for 6 or 24 h. LPS enhanced expression of HK2, PFKP and PKM2 at 6 h and this was inhibited by SIRT1 knockdown, demonstrating a direct impact of SIRT1 on the rate‐limiting enzymes of glycolysis. These data show a dynamic ‘metabolic shift’ in endothelial cells toward glycolysis from oxidative phosphorylation that is impaired when SIRT1 is reduced, findings similar to the role of SIRT1 in strokes.^9^ With regards to sepsis, this ‘metabolic shift’ has been observed in leukocytes and in septic patients and is thought to be a key regulator of effective host defence to pathogen challenge.^10^

In conclusion, we demonstrate a potentially important role for SIRT1 in the endothelial pathology of sepsis. Endothelial SIRT1 expression was increased by LPS and inhibition or reduction of SIRT1 impaired endothelial‐mediated vasodilation and monolayer integrity. As SIRT1 is known to be inactivated by oxidation and may be vulnerable in the setting of endothelial redox stress that is associated with sepsis, this upregulation may be in response to oxidant stress.^11^ Additionally, altered SIRT1 expression was associated most strongly with changes in FOXO1, as well as eNOS and TFAM, but not PGC1α, suggesting modulation of specific metabolic proteins by SIRT1 during sepsis. Metabolic changes were further shown by a LPS‐induced ‘metabolic shift’ from oxidative phosphorylation to glycolysis as reflected by upregulation of key glycolytic enzymes, which were mitigated by SIRT1 reduction. Thus, SIRT1 may coordinate an enhancement of glycolysis in order to meet rapidly increasing energy demands during sepsis and preserve endothelial function. Additional studies will be needed to define the role of metabolism in endothelial health during acute stress.

## CONFLICT OF INTEREST

None declared.

## FUNDING

This work was supported by a grant from the National Institute of General Medical Sciences at the National Institutes of Health (R35 GM138191 to RJS).

## Supporting information

Supporting Information
