# Supplementary material for: Loss of Sirtuin 1 (SIRT1) potentiates endothelial dysfunction via impaired glycolysis during infectious challenge
Source: Clin Transl Med. 2022 Sep 14;12(9):e1054. doi: 10.1002/ctm2.1054 (PMC9473483; doi:10.1002/ctm2.1054)
Supplement: Supplementary file 1 — Supporting Information [file CTM2-12-e1054-s001.docx]

Loss of Sirtuin 1 (SIRT1) Potentiates Endothelial Dysfunction via Impaired Glycolysis During Infectious Challenge.

Ryan J. Stark M.D.^1^*, Stephen R. Koch^1^, Cody L. Stothers M.D., Ph.D.^1^, Allison Pourquoi M.S.^1^, Celia K. Lamb^1^, Michael R. Miller M.D., Ph.D.^1^, Hyehun Choi Ph.D.^1^

Department of ^1^Pediatrics, Vanderbilt University Medical Center, Nashville, TN 37232.

Corresponding author (*):

Ryan Stark, M.D.

2200 Children's Way

5121 Doctors' Office Tower

Nashville, TN 37232-9075

Phone: (615) 875-8771

Email: ryan.stark@vumc.org

Short Title: SIRT1 loss impairs endothelial function during infection

Keywords: LPS, sepsis, endothelial, SIRT1, mitochondria

**METHODS:**

**Cells and culture:**

Pooled neonatal dermal human microvascular endothelial cells (HMVECs) were purchased from Lonza (Basel, Switzerland). HMVECs were grown in Endothelial Growth Media-2 (Lonza) supplemented with 5% fetal bovine serum (FBS). Cells were plated at a density of approximately 30,000 cells/cm2 and grown to confluence. Experiments were conducted between the 2nd and 5th passages. Media was exchanged at least every 3 days.

**Animals:**

Male C57BL/6 mice (Jackson Laboratory, Bar Harbor, ME), 12 to 15 weeks of age, were used. The animals were housed on a 12 hrs light/dark cycle and fed a standard chow diet with water ad libitum. Prior to vessel harvesting, animals were euthanized by placement in a sealed chamber with 100% CO_2_ provided at a flow rate of 2 L/min for 3 min. At that time, animals were monitored till they had > 1 min of respiratory cessation after which they underwent cervical dislocation. All procedures were performed in accordance with the Guiding Principles in the Care and Use of Animals, approved by the Vanderbilt University Institutional Animal Care and Use Committee.

**Transcript profiles in septic patients:**

Gene array profiles of blood samples were obtained from the publicly available National Centers for Biotechnology Information (NCBI) Gene Expression Omnibus (GEO) database. Values were obtained from GEO Dataset GSE26378 initially collected by Wong HR, et al. “Expression data from validation cohort of children with septic shock” ^1^ performed on a Affymetrix Human Genome U133 Plus 2.0 array and GSE134364 collected by Scicluna BP, et al “Protein-coding and non-coding RNA landscape in critically ill patients with sepsis”^2^ performed on a [HTA-2_0] Affymetrix Human Transcriptome Array 2.0. The databases were queried for SIRT1, TFAM, PGC1α, FOXO1, eNOS (NOS3), hexokinase 2 (HK2), phosphofructokinase-platelet (PFKP), pyruvate kinase M2 (PKM2), cytochrome C (CYCS) and ATP synthase F_0_ (MTATP6) and relative expression values were normalized to patient controls.

**Agonist and inhibitor reagents:**

The following reagents and concentrations were used in experiments: 0.1 μM EX527 (ActiveMotif, Carlsbad, CA, USA) and 100 ng/ml Ultra-Pure LPS (List Biological Laboratories, Campbell, CA, USA). Vehicle controls were either DMSO (<1%) for EX527 or PBS for LPS. Agonists, inhibitors, and vehicle controls were provided to cells in the presence of culture media.

**RNA isolation and real-time polymerase chain reaction:**

RNA was isolated from cultured cells 6 hrs and 24 hrs after LPS exposure via GenElute Mammalian Total RNA Miniprep Kit (MilliporeSigma, St. Louis, MO, USA) following the manufacturer’s instructions. Afterwards, 1.5 µg of total RNA was reverse transcribed into cDNA using the High Capacity cDNA Reverse Transcription Kit (Applied Biosystems, Life Technologies, Grand Island, NY, USA). Efficiency of the PCR reactions was tested by amplification of the target from serially diluted cDNA generated from reverse transcription to achieve an efficiency of 95% + 5%. Real-time PCR was performed using the TaqMan Fast Advanced Master Mix on a StepOnePlus Real Time PCR System (Applied Biosystems, Life Technologies). PrimeTime^®^ qPCR 5' Nuclease Assays (Integrated DNA Technologies, Inc., Coralville, IA, USA) were used to amplify the target mRNAs containing the following sequences: 5’-AATACCATCCCTTGACCTGAAG and 5’-GTGAAAGTGATGAGGAGGATAGAG for SIRT1; 5′-ACATCGCTCAGACACCATG and 5′-TGTAGTTGAGGTCAATGAAGGG for GAPDH.  Data were normalized as a ratio of threshold cycle of target mRNA to GAPDH and corrected for efficiency using the StepOne software.

**Transendothelial electrical resistance (TEER) assay:**

TEER analysis was performed utilizing the CellZScope2 (nanoAnalytics GmbH, Münster, Germany). HMVECs were plated on ThinWell Cell Culture inserts (0.4μm pore diameter, Greiner Bio-One, Monroe, NC, USA), coated with poly-L-lysine (2x) for 20 min then washed with Dulbecco’s phosphate-buffered saline (D-PBS). Inserts were then coated with glutaraldehyde 50% (1000x) for 15 min, washed with D-PBS, and lastly coated with gelatin (40x), then washed again with D-PBS. Afterwards, 70% ethanol was applied for 30 min followed by a 30 min wash in medium and then removed. Cells were plated at 40,000 cells/transwell and allowed to grow over 24 hrs until resistance stabilized. In some experiments, cell under 48 hrs of siRNA exposure prior to seeding. In addition, in some experiments, cells were pre-treated with EX527 inhibitor for 30 min prior to LPS. TEER was measured at 1 hr intervals. Data were normalized to the resistance prior to LPS challenge. Area under the curve was then calculated as the total combined increase in resistance (positive inflection) with the decrease in resistance (negative inflection) relative to pre-LPS resistance normalized to 100%. Averages for AUC with 95% CI were then calculated and compared per statistical analysis methods.

**Vascular Reactivity Measurements:**

Mesenteric resistance arteries (first order branches of the superior mesenteric, ∼100 μM ID) were excised, cleaned of fat and connective tissues, and cut into 2-mm-length rings in an ice-cold physiological salt solution (PSS) consisting of the following (in mM): 130 NaCl, 4.7 KCl, 1.18 KH2PO4, 1.18 MgSO4·7H2O, 1.56 CaCl2·2H2O, 14.9 NaHCO3, 5.6 glucose, and 0.03 EDTA. Mesenteric rings were incubated in Dulbecco's modified Eagle medium (DMEM) containing LPS (100 ng/ml, 24 hrs). EX527 (0.1 μM) or vehicle control (0.01% DMSO) were applied 30 min prior to LPS exposure. Rings were subsequently mounted in wire myographs (Danish Myo Technology A/S, Aarhus, Denmark) containing warmed (37°C), oxygenated (95% O2/5% CO2) PSS and allowed to equilibrate for at least 45 min under a passive force of 2 mN. Afterwards, arterial integrity was assessed by stimulation of vessels with 120 mM KCl and, after contraction, reached a plateau, and the rings were washed. Subsequently, the rings were stimulated with phenylephrine (PE; 10^-6^ M), followed by relaxation with acetylcholine (ACh; 10^-6^ M). More than an 80% relaxation response to ACh was taken as evidence of an intact endothelium. Endothelium-dependent relaxation was performed on PE-contracted (10^-6^ M) rings by cumulative addition of ACh (10^-8^ to 10^-4^ M), and endothelium-independent relaxation was tested using sodium nitroprusside (SNP; 10^-9^ to 10^-5^ M). Data was normalized prior to the addition of ACh or SNP as a percentage of the contraction by PE in each ring.

**Western blot and electrophoresis:**

Cell lysates were collected at 6 or 24 hrs (as indicated) after LPS exposure. Protein extracts (50μg/sample) were separated by SDS electrophoresis on a polyacrylamide gel (10%) and transferred to nitrocellulose membranes. Membranes were blocked with Odyssey Blocking Buffer (LI-COR Biosciences, Lincoln, NE, USA) for 1 hr at room-temperature. Membranes were incubated with primary antibodies overnight at 4 °C on a rocker. Antibodies were as follows: SIRT1 (Novus Biologicals, Centennial, CO, USA), p-FOXO1, FOXO1, PGC1α, TFAM, eNOS, p-p38, p38, p-JNK, JNK, p-ERK, ERK, HK2, PKFP, PKM2 and α-tubulin (Cell Signaling Technology, Danvers, MA, USA). Membranes were then incubated with fluorescent secondary antibodies and analyzed on the Odyssey Imaging System (LI-COR Biosciences). Protein quantification was performed via densitometry and normalized as a ratio of phosphorylated protein to respective total protein or total protein to tubulin.

**Immunoprecipitation (IP)**

HMVECs were lysed with lysis buffer (0.01M Tris base, 1mM EDTA, 30mM NaCl, 1% Nonidet P-40, protease inhibitor cocktail, and phenylmethylsulfonyl fluoride (PMSF) at pH 7.4) for 1 hr with nutation at 4ºC, and centrifuged for 30min at 20,000g. Supernatants (1 mg of protein) were incubated with α-SIRT1 antibody (10 µg, Novus #NBP1-51641) or mouse IgG control (10 µg, Cell Signaling Technology) for 1.5 hrs, then incubated with Dynabeads protein-G (Invitrogen, #10003D) for 1 hr with nutation at 4ºC. Beads were washed with lysis buffer, resuspended in SDS sample buffer, and boiled. The associated proteins were then analyzed by western blot as above.

**siRNA transfection:**

HMVECs were treated with siRNA (scrambled siControl, siSIRT1) according to the manufacturer’s recommendations. In brief, siRNA was procured from Dharmacon (Lafayette, CO, USA). siRNA (25 nmol/L) was incubated with Dharmafect (Dharmacon) in serum-free medium for 20 min. The resultant complex of siRNA-Dharmafect was added to the cells in 5% FBS media without antibiotics for 6 hrs. Afterwards, the transfection media was replaced with complete media including antibiotics for another 66 hrs for a total of 72 hrs siRNA incubation time prior to agonist exposure or imaging.

**Cytokine and chemokine production:**

Supernatants from cell cultures were collected at 6 hrs post-LPS exposure and stored at -80 °C until analyzed. Interleukin (IL)-6 (eBioScience, San Diego, CA, USA), angiopoeitin-2 (Ang-2, Boster Bio, Pleasanton, CA, USA), granulocyte colony stimulating factor (G-CSF), soluble vascular cell adhesion molecule (sVCAM), vascular endothelial growth factor (VEGF) and IL-8 (R&D Systems, Minneapolis, MN, USA) concentrations were assessed using commercially available enzyme-linked immunosorbent assay (ELISA) kits according to the manufacturer’s specifications.

**Proximity ligation assay (PLA):**

Proximity ligation assays were performed using a commercially available kit according to the manufacturer’s instructions (Duolink PLA, MilliporeSigma, St. Louis, MO, USA). Briefly, 96-well glass bottom plates were pretreated with poly-L-lysine, glutaraldehyde 50% and gelatin in the same concentrations and times as described for TEER. Cells were exposed to LPS for 6 hrs and then the cells were washed with PBS and exposed to 4% formaldehyde fixative in PBS for 15 min then permeabilized with 0.1% TritonX-100 in PBS for 15 min. Following solution removal, 40 μL of Duolink blocking solution (MilliporeSigma) was placed in each well and incubated at 37 °C for 1 h. The blocking solution was then replaced with Duolink antibody diluent containing the following primary antibodies at a 1:100 dilution: eNOS rabbit mAb (clone #D9A5L, Cell Signaling Technology, Danvers, MA, USA), SIRT1 mouse mAb (clone # 834918, Novus Biologicals, Centennial, CO, USA), FOXO1 rabbit mAb (clone # C29H4, Cell Signaling Technology) and TFAM rabbit mAb (clone #DFH8, Cell Signaling Technology). Plates were then sealed and incubated overnight at 4 °C. The next day, the primary antibodies were removed, and the cells were washed with Duolink Wash Buffer. Duolink PLA probes (anti-rabbit secondary antibody with plus oligonucleotides and anti-mouse secondary antibody with minus oligonucleotides) were diluted in a 1:5 ratio in Duolink Antibody Diluent and 40 μL of probe containing solution was added to each well for 1 h at 37 °C. Kit ligase was added at a 1:40 dilution to Duolink Ligation Buffer, which contained pre-mixed concentrations of bridging oligonucleotides, and added to each well for 30 min at 37 °C. Next, cells were washed and kit-provided polymerase was added in a 1:80 dilution in Duolink Amplification Buffer and applied to each well for 100 min at 37 °C. Duolink Orange fluorescent probes (ex 554/em 576) were present in the amplification buffer. Cells were then washed and Duolink Wash Buffer containing BioTracker 488 (ex 500/em 515, MilliporeSigma) per mL of buffer was added to each well at room temperature for 10 min. The wash buffer was then removed and replaced with 120 μL of Live Cell Imaging Solution (Thermo Fischer Scientific) and imaged at the appropriate wavelength using an inverted confocal microscope (Zeiss LSM 880, Carl Zeiss AG, Oberkochen, Germany). Fluorescent reactions and cell number were counted by averaging two random fields per well using ImageJ software (National Institutes of Health, Bethesda, MD, USA).

**Mitochondrial oxygen consumption and glycolysis assay:**

Cells were plated in a 96-well Seahorse assay plate at 12,000 cells/well in Seahorse Assay Media and assessed on the Seahorse XFe 96 Extracellular Flux Analyzer (Agilent Technologies, Santa Clara, CA, USA). For the glycolysis stress test, cells were sequentially treated with 10 mM glucose (RPI, Mount Prospect, IL, USA), 1 mM oligomycin (Agilent Technologies), and 50 mM 2 deoxyglucose (2-DG) (MilliporeSigma). For the mitochondrial stress test, assay media were supplemented with 10 mM glucose, and cells were sequentially treated with 1 mM oligomycin (Agilent Technologies), 1 mM FCCP (Agilent Technologies), and 0.5 mM of antimycin A and rotenone (Agilent Technologies).

**Statistical analysis:**

For cell culture experiments, data are expressed as means ± SE of multiple, individual experiments. Comparisons of treatment groups, controls, and other conditions were done via unpaired t-test for single comparisons and one-way ANOVA, with Tukey correction, for multiple-group comparisons. For RNA transcript expression of blood samples, data are expressed as medians ± 75% interquartile range for each data subset. Comparisons of groups were done via a Mann-Whitney test and Spearman correlation for direct expression comparison with 95% CI. For vascular reactivity studies, concentration-response curves were fitted using a nonlinear curve and two pharmacological parameters were obtained: the maximal effect by the agonist (Emax) and EC50 (molar concentration of agonist producing 50% of maximum response). All analysis was done using GraphPad Prism 9 statistical software (GraphPad Soft-ware Inc., La Jolla, CA USA). A p-value cutoff of < 0.05 was used for statistical significance.

**REFERENCES:**

1. Wong, H.R.*, et al*. Genomic expression profiling across the pediatric systemic inflammatory response syndrome, sepsis, and septic shock spectrum. *Crit Care Med*. 2009; 37: 1558-1566.

2. Scicluna, B.P.*, et al*. The leukocyte non-coding RNA landscape in critically ill patients with sepsis. *Elife*. 2020; 9:

**SUPPLEMENTAL DATA:**

**
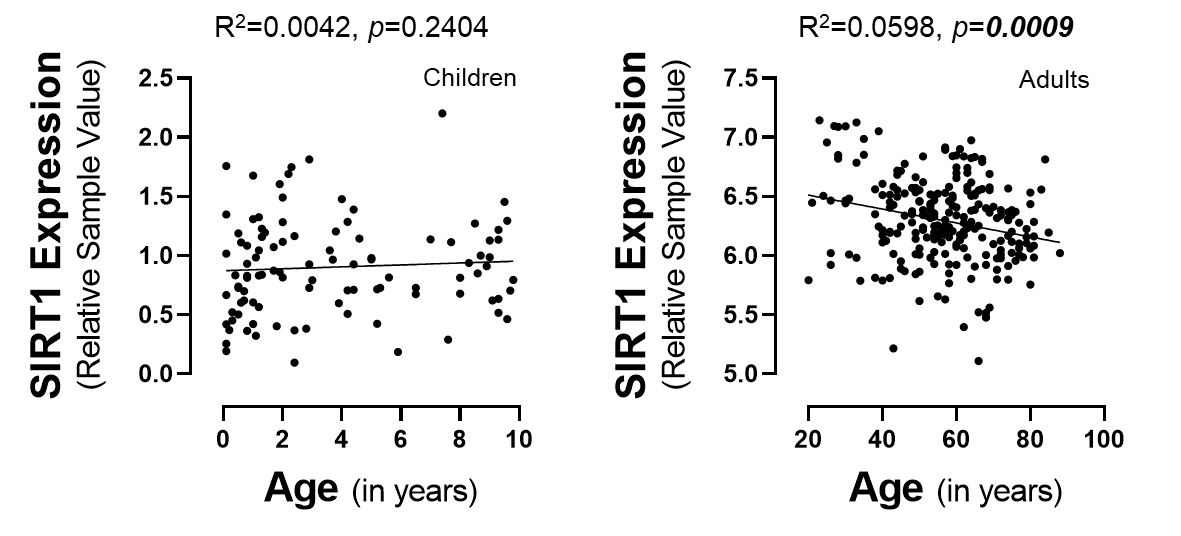
**

**Supplemental Figure 1:** Expression values for SIRT1 transcript matched to patient age for children (*left,* GSE26378) and adults (*right,* GSE134364) within the respective cohorts. Associated coefficients of determination (R^2^) and linear regression are shown with Spearman r correlation coefficient *p* values.


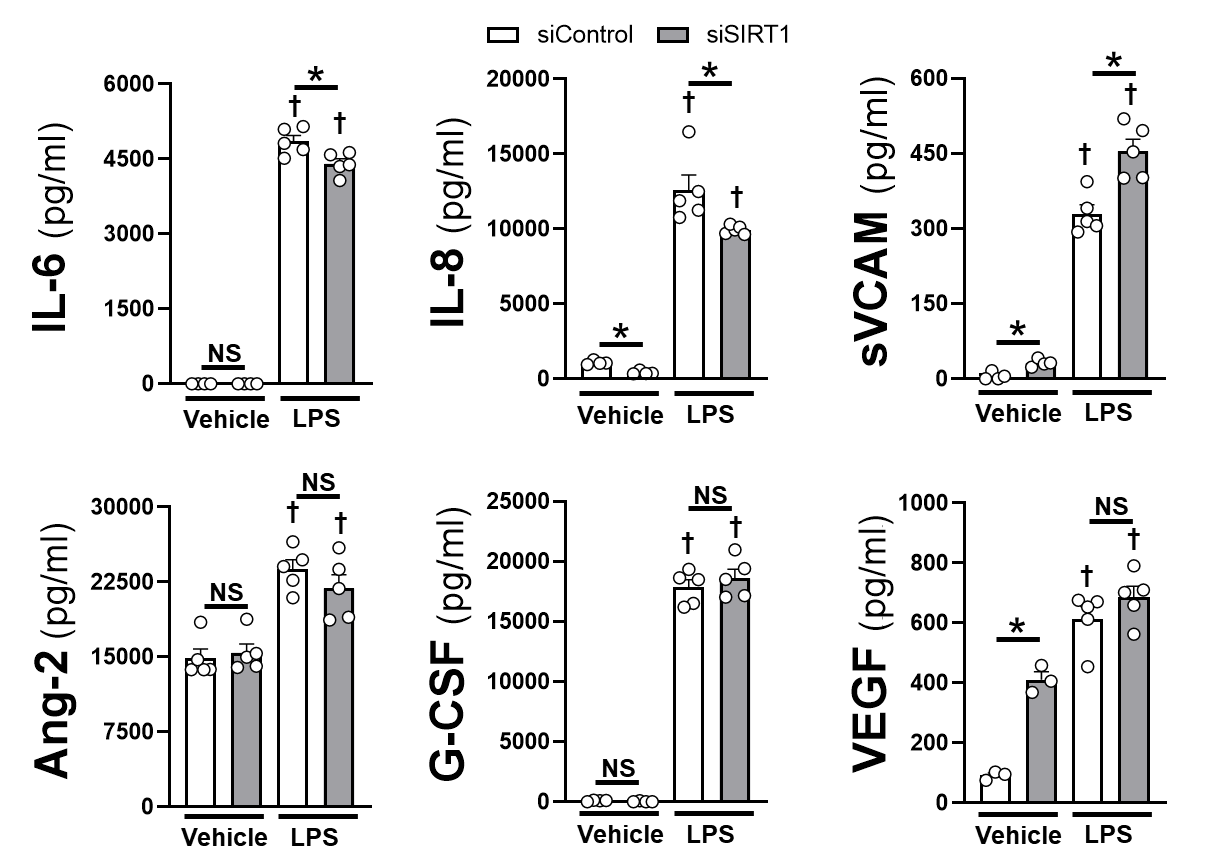


**Supplemental Figure 2:** Cytokine concentrations (IL-6, IL-8, sVCAM, Ang-2, G-CSF, VEGF) shown in their respective concentrations in HMVEC cells treated with siControl or siSIRT1 72 hours prior to exposure to subsequent LPS (100 ng/ml) stimulation for 6 hours versus vehicle control. n=3-6 individual replicates per group. * = *p* < 0.05 between designated groups. † = *p* < 0.05 between indicated group and respective siRNA vehicle control. NS = non-significant.

**
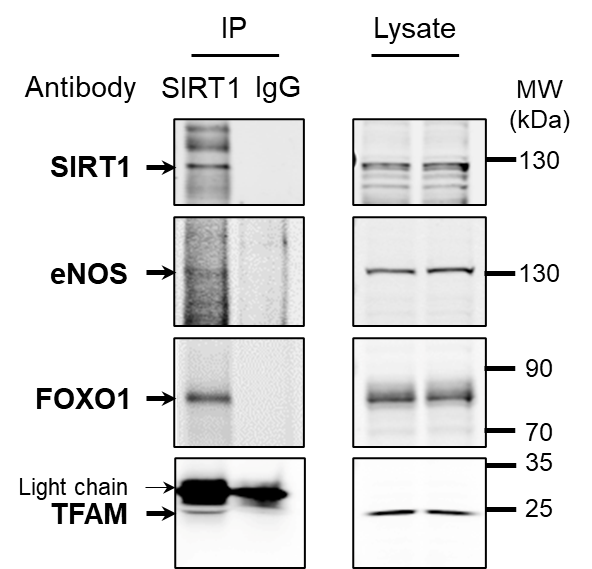
**

**Supplemental Figure 3:** Representative images of SIRT1, eNOS, FOXO1 or TFAM in untreated HMVECs immunoprecipitated with anti-SIRT1 antibody or IgG controls (*left*) with associated cell lysates (*right*).

**
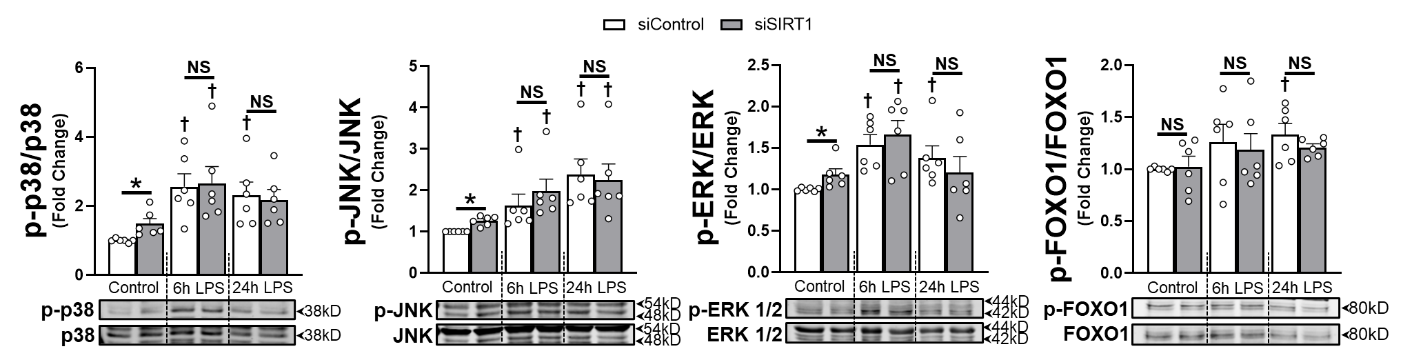
**

**Supplemental Figure 4: A)** Densitometry analysis and associated images of whole cell lysate western blots (n=6 individual replicates per group) calculated for the indicated MAPK proteins (p38, c-jun N-terminal kinase, JNK, extracellular signal-regulated kinase 1/2, ERK 1/2) and FOXO1. Phosphorylation signal is normalized to total non-phosphorylated protein within each analysis. HMVECs were treated with respective siRNA for 72 hrs prior to LPS exposure (100 ng/ml) at the times indicated. * = *p* < 0.05 between designated groups. † = *p* < 0.05 between indicated group and respective siRNA vehicle control. NS = non-significant.


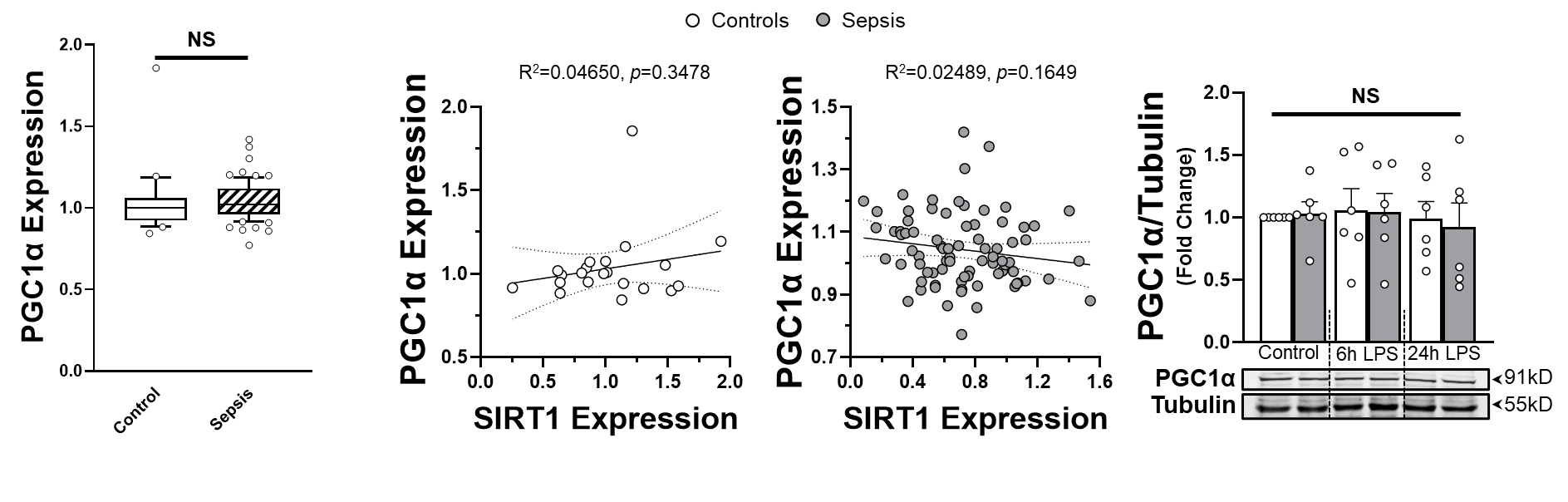


**Supplemental Figure 5:** **A)** PGC1α mRNA transcript relative values are shown for children with or without sepsis from the respective GEO Dataset (GSE26378). The median expression value is shown with a horizontal bar per condition with 75% interquartile range normalized to the respective cohort controls. **B)** Expression values for PGC1α in comparison to individually matched SIRT1 transcript values for control patients versus those with sepsis. R^2^ values with Spearman r correlation associated *p* values are displayed. Linear regression (solid lines) with 95% CI (dotted lines) are shown. **C)** Densitometry analysis and associated images of whole cell lysate western blots (n = 6 individual replicates per group) calculated for total PGC1α normalized to tubulin in HMVECs treated with respective siRNA for 72 hrs prior to LPS exposure (100 ng/ml) at the times indicated. NS = non-significant.


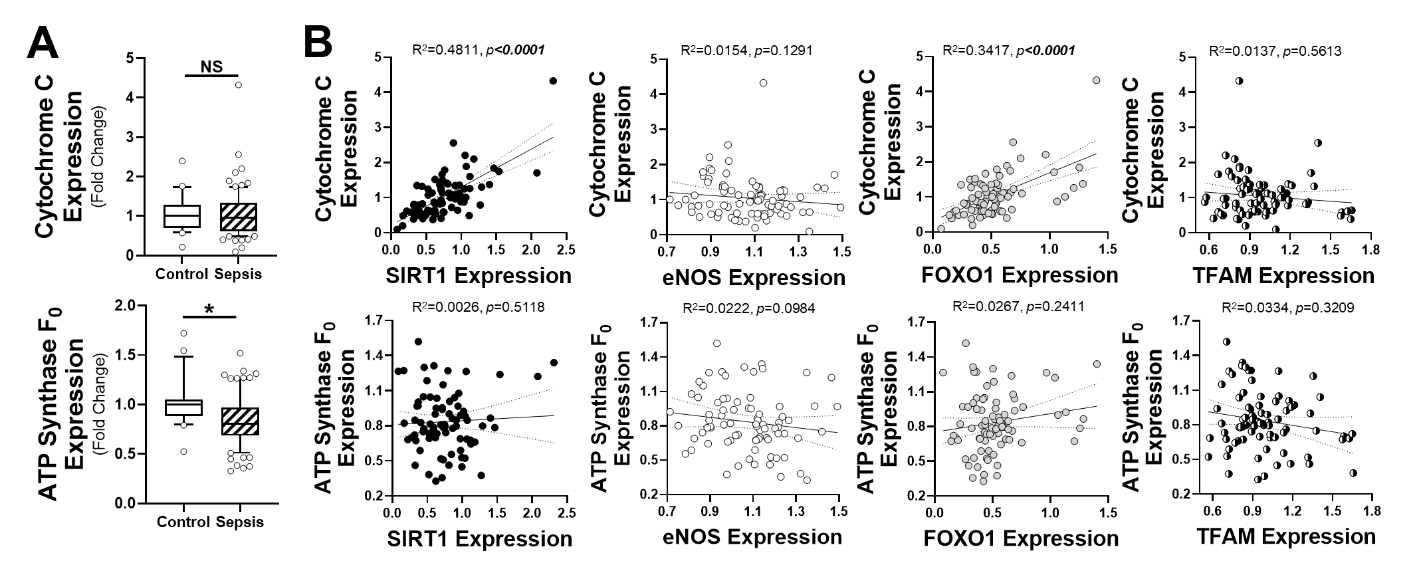


**Supplemental Figure 6: A)** Cytochrome C or ATP synthase F_0_ mRNA transcript relative values are shown for children with or without sepsis from the respective GEO Dataset (GSE26378). The median expression value is shown with a horizontal bar per condition with 75% interquartile range normalized to the respective cohort controls. **B)** Expression values for cytochrome C or ATP synthase F_0_ in relation to individually matched transcript levels for SIRT1, eNOS, FOXO1 or TFAM for patients with sepsis. Associated coefficients of determination (R^2^) are shown with Spearman r correlation coefficient *p* values. Linear regression (solid lines) with 95% CI (dotted lines) are displayed. * = *p* < 0.05 between designated groups. NS = non-significant.
